# Advancements in Performance Monitoring: A Systematic Review of Sensor Technologies in Rowing and Canoeing Biomechanics

**DOI:** 10.3390/sports12090254

**Published:** 2024-09-13

**Authors:** Maria I. Cruz, Hugo Sarmento, Ana M. Amaro, Luís Roseiro, Beatriz B. Gomes

**Affiliations:** 1 University of Coimbra, Research Unit for Sport and Physical Activity (CIDAF), Faculty of Sports Sciences and Physical Education, 3040-248 Coimbra, Portugal; hgsarmento@gmail.com (H.S.); beatrizgomes@fcdef.uc.pt (B.B.G.); 2 University of Coimbra, CEMMPRE-ARISE, Department of Mechanical Engineering, 3030-788 Coimbra, Portugal; ana.amaro@dem.uc.pt (A.M.A.); lroseiro@isec.pt (L.R.); 3 Coimbra Institute of Engineering, Polytechnic University of Coimbra, Rua Pedro Nunes—Quinta da Nora, 3030-199 Coimbra, Portugal; 4 Research Centre for Natural Resources Environment and Society (CERNAS), Polytechnic University of Coimbra, Bencanta, 3045-601 Coimbra, Portugal

**Keywords:** sport biomechanics, data collection, oar sports, paddlesports, optimization

## Abstract

A comprehensive understanding of sports biomechanics is essential for optimizing athletic performance. Recent advancements in sensor technology, particularly inertial sensors, have transformed the landscape of sports performance analysis. These sensors offer profound insights into the kinematic and kinetic aspects of sports, with a particular impact on water-based sports such as rowing and canoeing. This systematic review aims to establish a comprehensive framework for examining sensor technologies and evaluating biomechanical performance in rowing and canoeing. The authors systematically searched four prominent databases (Web of Science, Scopus, Science Direct, and Sage Journals), concentrating on research that has employed sensors to analyze critical performance variables in rowing and canoeing. Our exclusion criteria included manuscripts that exclusively addressed ergometer-based studies, those lacking sensor-related content, unrelated subjects, and publications dating back more than 15 years. The authors used the National Heart, Lung, and Blood Institute Quality Assessment Tools to assess study quality and bias risk. A total of 11 studies were included in this review. This review also acknowledges the limitations, such as the exclusion of gray literature and studies in languages other than English, which may have limited the scope of the research. The studies were synthesized qualitatively, focusing on key variables, including oar/paddle force, boat speed, and technique, and were analyzed, providing quantitative insights. Sensor technology has ushered in a new era of rowing and canoeing performance analysis.

## 1. Introduction

Sports performance monitoring encompasses various dimensions, including biomechanical, physiological, and psychological aspects. Biomechanical analysis focuses on understanding human movement and improving technique [1,2], while physiological monitoring assesses parameters such as heart rate, oxygen consumption, and muscle activation, which directly impact performance [3]. Psychological monitoring assesses mental factors such as stress, motivation, and concentration, which are critical for optimal performance [4,5].

To bridge the gap between these multifaceted performance metrics and practical applications, recent technological advancements have allowed for specific feedback in sports, which is crucial for coaches and athletes to improve their performance and prevent injuries [6]. Data obtained from training and competition have become more reliable and objective [7]. Traditionally, athlete performance assessments have been conducted in a controlled laboratory setting using various tests. However, this approach may only partially capture the specificity of the training and monitoring. For instance, although on-water rowing [8]/canoeing [9] and ergometer simulations share similarities, significant biomechanical differences can affect motor control patterns and techniques, suggesting that ergometer training cannot replace the on-water experience.

Significant progress in microelectronics and other microtechnologies has enabled the development of testing and monitoring systems for elite athletes in many sports, specifically designed to test in real-sport workout conditions [10]. Inertial measurement units (IMUs) incorporate these technologies, which measure acceleration and angular velocity along three perpendicular axes and indirectly measure specific forces based on the laws of motion. Inertial sensors can combine with sensors like strain gauges, GPSs, or potentiometers to monitor an athlete’s biomechanical performance in various settings like in a laboratory, training environment, or competition [11].

Previous studies have used sensors to monitor sports performance [12,13,14]. However, in water sports like canoeing and rowing, sensor technology requires adaptations to overcome the challenges posed by the aquatic environment [15,16]. Specialized sensor designs and waterproofing mechanisms are essential to ensure accurate data collection in wet conditions. It is recommended to use multiple types of sensors and correlate the collected variables when monitoring athletes during aquatic training [16]. This approach enables a comprehensive assessment of performance and technique, providing insights into stroke mechanics, power generation, movement patterns, and course optimization. By leveraging various sensors and correlating variables, a detailed understanding of an athlete’s performance in water sports can be achieved, leading to targeted improvements in training and performance.

While existing systematic reviews have demonstrated the reliability, validity, and utility of inertial sensors in general sports applications [11], a specific overview of rowing and canoeing and their implementation is still needed to improve performance analysis. While previous reviews have focused on ergometer performance [8], injuries [9,10], and metabolism [17], there appears to be a gap in exploring the combined use of sensors in these sports.

Therefore, this review aims to systematically examine the use of sensor technologies in sports biomechanics, focusing on rowing and canoeing, to identify and evaluate the kinetic, kinematic, and dynamic parameters measured, the sensors employed on paddle and oar, and their data transmission frequencies, storage solutions, and data analysis approaches, thereby contributing to the optimization of performance monitoring in these aquatic sports. By systematically reviewing and synthesizing existing research, this study intends to provide a comprehensive framework for sensor utilization in these sports, thereby enhancing training effectiveness, performance, and injury prevention strategies.

## 2. Methods

### 2.1. Search Strategy and Study Selection

**Figure A1:**
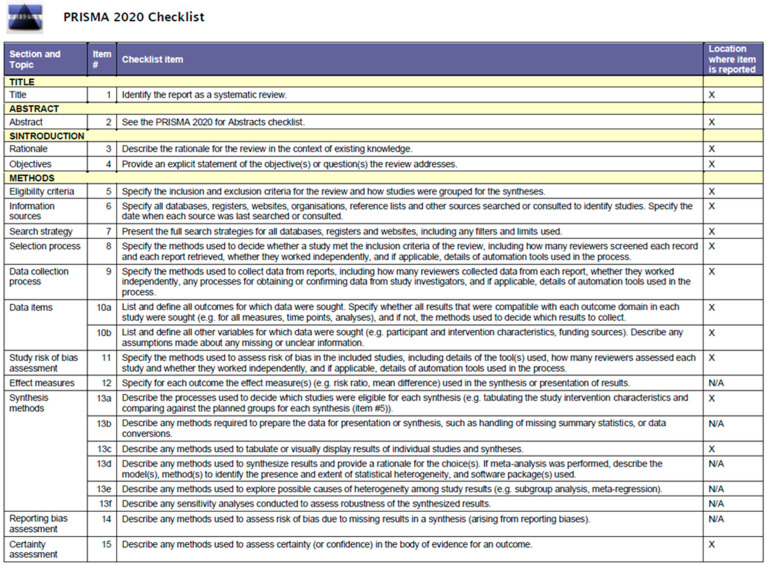

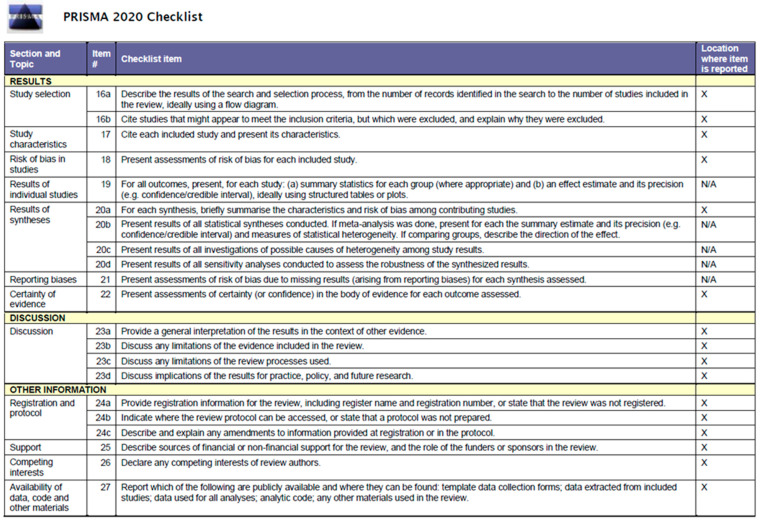
Prisma 2020 checklist adapted from [36].

A systematic review was conducted on the 20th of January 2023, following the Preferred Reporting Items for Systematic Reviews and Meta-Analyses (PRISMA) criteria, utilizing the PRISMA-ScR extension for reporting the findings of systematic reviews [18]. To ensure the accessibility of the methodology and enhance the integrity of the review process, the protocol was prospectively registered on INPLASY under the registration number INPLASY202390044 with the DOI: 10.37766/inplasy2023.9.0044. The PRISMA checklist is available in the Appendix A (Figure A1).

Before identifying the relevant journal manuscripts, the authors of this paper undertook the task of determining the search keywords. The databases were selected for their relevance and scope in sports science. The searches were carried out using the Web of Science Core Collection, Scopus, Sage Journals, and ScienceDirect, using the following keywords: (“athlete$ row*” OR “athletes canoe*” OR canoeing OR rowing) AND (“inertial sensor$” OR sensor$ OR gyroscope OR meter OR “strain gauge$” OR GPS OR “Global Position System” OR GNSS OR “Global Navigation Satellite System”) AND (kine OR velocity OR power OR tim* OR force$ OR angle$ OR *feedback). The search strategy was adapted for each database, employing specific combinations of Boolean operators and keywords (Appendix A—Table A1). Filters were applied to limit the results to the specified publication period and language, and all search strings were carefully documented to ensure replicability. All retrieved manuscripts were compiled into a reference manager (EndNote X20, Thomson Reuters, Philadelphia, PA, USA) for further analysis upon completion of the search. At this stage, some manuscripts were excluded due to duplication or other reasons, resulting in a total of 1180 manuscripts identified and screened for eligibility. 

The criteria for inclusion and exclusion were based on the PECO framework, which stands for Population, Exposure, Comparator, and Outcome, detailed in Table 1. The inclusion criteria required that manuscripts be written in English, reference the use of sensors, employ sensors to measure variables related to paddle or oar activities, and use sensors exclusively for outdoor measurements. The exclusion criteria specified that manuscripts not published in scientific journals, those that did not establish a clear relationship or relevance to the research question, and those published before 2008 were to be excluded, considering more outstanding technological advances after that.

### 2.2. Screening Process

Two authors (MC and BG) meticulously conducted the screening process. They independently reviewed the retrieved records, including titles and abstracts. Subsequently, they individually assessed the full texts of the selected records and extracted the data from the selected studies on a Microsoft Excel sheet. When discrepancies were identified, a collaborative reevaluation was conducted to reach a consensus. In cases where a consensus could not be achieved, a third author (AM) made the final decision. No contact was made with the authors of the included studies for data confirmation or clarification, as all the necessary information was available in the published reports.

### 2.3. Quality of the Studies and Extraction of Data

In this systematic review, two main categories of findings have emerged. The first category focuses on variables and sensors, offering an overview of the types of variables collected and the sensors used for data collection. The second category, labeled “Data Collection, Processing, and Interface”, explores the key aspects of the data collection process, including sampling frequency, data collection, analysis methods, and the type of interface employed to present variables to users.

The authors selected the National Heart, Lung, and Blood Institute (NHLBI) Quality Assessment Tools for Observational Cohort and Cross-Sectional Studies to ensure the reliability and validity of the studies included in our review of sports biomechanics and sensor technologies. These tools were chosen for their comprehensive nature and ability to provide a structured and detailed evaluation of methodological quality and risk of bias across various study designs, such as randomized controlled trials, cohort studies, case-control studies, and cross-sectional studies.

The NHLBI tools are particularly suited to our review because of their broad applicability and objective criteria, which are crucial for assessing studies in fields that combine complex technologies with human performance metrics. These tools allow for an in-depth analysis of critical aspects of study design, conduct, and reporting, including the robustness of the study design, participant selection strategies, accuracy of measurements, control of biases, consistency of data, adequacy of statistical analysis, clarity in result presentation, and the validity of discussions and conclusions. 

Moreover, the NHLBI tools’ final scoring system, categorizing the studies into “Good”, “Fair”, and “Poor”, offers a clear, intuitive assessment of each study’s methodological quality. This grading facilitates informed judgments about the relevance and weight of the studies’ findings in our review. Their significance and objectivity thus drove the choice of the NHLBI Quality Assessment Tools and the detailed guidance they provide, ensuring that our systematic review is based on methodologically sound and reliable research.

The quality assessment process involved two independent authors, MC and BG, who individually evaluated each included study using the NHLBI Quality Assessment Tools. In cases of disagreement between the two authors, consensus discussions were employed to resolve any discrepancies. This rigorous assessment process allowed us to evaluate the overall methodological quality and risk of bias in each study, ensuring the reliability of the evidence incorporated into our review.

In parallel with the quality assessment, a structured data extraction process was conducted to systematically gather pertinent information from the selected studies. This process involved systematically collecting data related to variables, sensors, data collection methods, processing techniques, and the type of interface used to display variables to users. 

## 3. Results

### 3.1. Search Results

**Figure 1:**
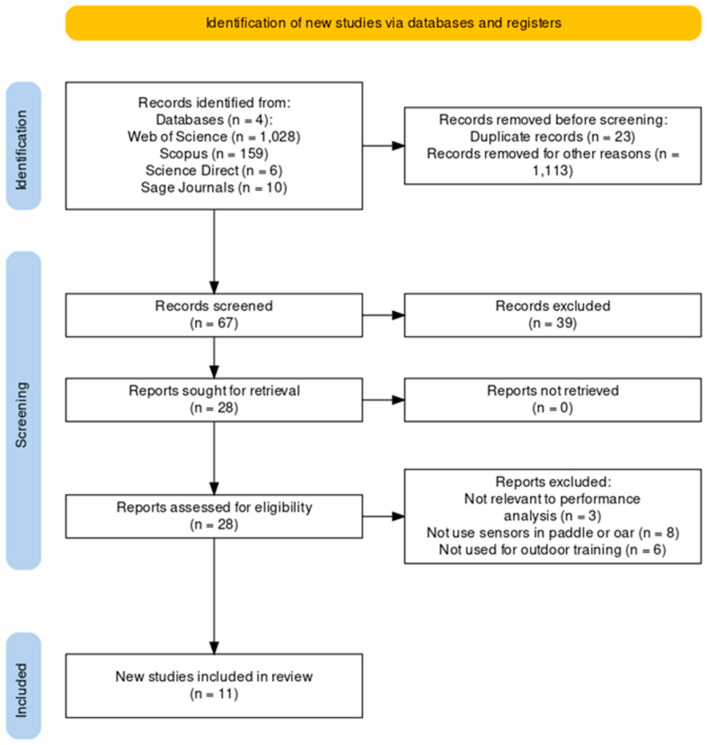
Prisma Flow Diagram.

After a comprehensive search, 1203 manuscripts were initially identified from electronic databases and the references of reviewed manuscripts (Figure 1). Following a rigorous screening process that involved removing duplicates and evaluating titles, publication types, and other criteria, 1136 manuscripts were excluded. Subsequently, 67 manuscripts were deemed suitable for abstract analysis. From this subset, 28 manuscripts were selected for an in-depth full-text examination.

During the full-text assessment, 17 manuscripts were found to be unsuitable for inclusion based on specific criteria. More precisely, three manuscripts did not pertain to performance analysis, eight did not involve the use of sensors in paddle or oar applications, and six were unrelated to outdoor training. As a result, these 17 manuscripts were excluded from further consideration. After this, thorough a screening and selection process, 11 studies were deemed eligible and included in this comprehensive review.

The included studies varied in their designs and methodologies, and the differences were qualitatively synthesized to provide insights into the use of sensors in rowing and canoeing.

### 3.2. Results of NHLBI Quality Analysis 

The NHLBI Quality Assessment Tools consist of tailored criteria sets for different study types, including randomized controlled trials, cohort studies, case-control studies, and cross-sectional studies. These criteria address essential methodological aspects such as study recruitment, sampling methods, data collection procedures, statistical analyses, and results presentation. The authors adopted these tools to enhance the transparency and objectivity of our quality assessment process.

Our systematic review encompasses eleven studies, comprehensively exploring the relevant literature. Each study underwent a rigorous quality assessment aimed at evaluating methodological robustness and potential sources of bias.

The analysis revealed that 73% of the included studies received a “GOOD” rating, indicating high methodological quality with rigorous research designs, well-executed methodologies, and minimal risk of bias. In contrast, 27% of the studies were rated as “FAIR”, indicating moderate methodological quality. While these studies exhibited specific strengths, they also presented some limitations or potential sources of bias.

The authors have synthesized these findings in Table 2 to enhance the accessibility of the quality assessment outcomes. This table encapsulates the critical dimensions, including the bias risk assessment and the identified methodological strengths and weaknesses for each included study.

It is important to note that none of the studies were rated as ‘POOR’, suggesting that all the included studies had some degree of methodological rigor, although the level of rigor varied. 

### 3.3. Overview of Variables and Sensors

This section provides an overview of the variables collected and the sensors utilized for data collection. Collected variables refer to the data points or measurements obtained during the data collection process tailored to the research or study objectives. These variables may include numerical values, categorical data, textual information, or images. For a concise overview of the variables collected and the corresponding sensors used, refer to Table 3.

Table 3 presents systems developed in both the commercial and academic environments. The commercialized systems include the Kayak Power Meter [19,20], Peach Power Line [21,22], eKayak [23], and Vaaka [24]. In the academic context, systems have been developed by Kleshenev [25], Sturm et al. [26], Gomes et al. [27], Castro et al. [6], and Prétot et al. [28].

### 3.4. Presentation of Results: Data Transmission, Processing, and Visualization

The reviewed manuscripts were evaluated with regard to data transmission and processing. The methodology assessed sampling frequency, storage and transmission systems, data visualization, and analysis software, as summarized in Table 4.

**Figure 2:**
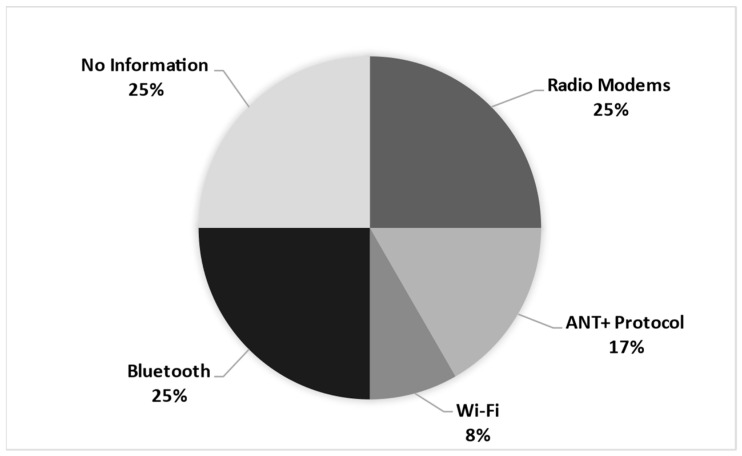
System to data transfer.

Figure 2 illustrates the distribution of the wireless data transfer technologies used across various systems. The data show that radio modems and Bluetooth are the most commonly used technologies, collectively representing 25% of usage. The ANT+ Protocol is also noteworthy, accounting for 17% of the technologies utilized. Despite its widespread recognition, only 8% of Wi-Fi instances are employed. Notably, 25% of the cases lacked sufficient information regarding the specific technology.

## 4. Discussion

The complexity and dynamic nature of rowing and canoeing biomechanics present significant challenges for performance optimization, necessitating advanced and precise monitoring technologies. Existing methods often fail to capture the full scope of motion and force dynamics, particularly in real-world aquatic environments. This research was conducted to address this gap by systematically reviewing and assessing the efficacy of various sensor technologies in these sports.

The primary aim of this study is to develop a comprehensive framework for selecting and utilizing sensors that effectively measure critical biomechanical parameters, thereby enhancing the performance analysis and training outcomes in rowing and canoeing.

### 4.1. Methodological Quality and Data Extraction of the Studies Analyzed 

The methodological quality of the studies is essential for the reliability of the review results. High-quality studies provide robust evidence, while moderate-quality studies require cautious interpretation. Some studies were rated as “fair” because they were conducted in laboratory settings, which may not fully replicate real-world scenarios.

This review examines all aspects of sensor technology, including sensor types and data transmission, focusing on methodology and practical application. Some studies were rated “fair” due to their limited relevance to specific sensor aspects. The subjective nature of certain criteria introduced some bias, which was addressed through team consensus discussions.

It is recommended that quality assessment guidelines be developed specifically for the diverse aspects of sensor technology. This will ensure methodological rigor and real-world applicability, improve study classifications, and advance sensor research.

### 4.2. Variables and Sensor Technologies Used in the Studies

Sensors are crucial for capturing and measuring data from the environment or specific objects. They are devices designed to monitor physical properties such as temperature, pressure, humidity, light intensity, motion, chemical composition, etc. These sensors are often integrated with data acquisition systems that are responsible for collecting, processing, and recording the data to facilitate subsequent analysis [7].

The variables collected in a given sport can significantly impact data analysis. Therefore, having adequate knowledge of the variables to be selected in a study is indispensable. In the case of rowing and canoeing, the number of variables that influence sports performance is vast. Many of these variables are interrelated, which can streamline data collection and reduce the number of sensors. 

To better understand the role of specific sensors in rowing and canoeing, the following table outlines some critical sensors used in these sports, the variables they measure, and their applications. Table 5 provides a clear overview of how each sensor contributes to performance analysis and optimization.

From the analyzed articles in this review, it is observed that many studies focus on similar variables, with the most common being the rowing or paddling force [6,20,21,22,25,26,27,28], boat speed and acceleration [6,20,21,22,23,27,28], and stroke rate [20,21,23,24,27]. It is important to note that this review primarily focused on implementing sensors in the boat, paddle, and oar, minimizing the analysis of sensors applied directly to athletes.

Various companies have developed and commercialized devices to measure specific variables in water sports. The Kayak Power Meter system by One Giant Leap in New Zealand includes multiple strain gauges and an inertial measurement unit (IMU) integrated into the paddle shaft, increasing its weight by approximately 150 g compared to a standard paddle [19,20,29]. The Peach Power Line by Peach Innovations (UK) attaches to the oar and the boat, adding 1 kg to the vessel. This device allows for rowing analysis without specifying the incorporated sensors and shows the low errors associated with the acquired variables [21,22,30].

The eKayak, developed for the Italian Olympic Committee, contains 9-axis IMU sensors, a high-sample-rate GPS device, and pairs of force sensors applied to the paddle and footrest. This prototype combines IMU sensors, GPS, and force sensors. The agent node of the paddle is fixed inside the shaft, comprising two pairs of strain gauges positioned on the right and left sides of the shaft. This node adds 30 g to the paddle’s weight, and the controller node implemented in the boat adds 450 g [27]. Other devices include the Vaaka kayak cadence, which uses a triaxial accelerometer to measure stroke rate. It is embedded in a waterproof box and implemented in the kayak paddle between the paddler’s hands [24,31].

Universities or individuals have developed systems such as Kleshnev’s system, which combines an accelerometer, potentiometer with strain gauge, and electromagnetic impeller [25]; the Kayak XL System, which has strain gauges, a gyroscope, and an accelerometer mounted on the paddle [26]; and the FPaddle System, which is composed of deformation sensors, a force transducer, and a triaxial accelerometer dedicated to the kayaking modality [27].

Studies also report combinations of the sensors developed, such as the system by Castro et al. [6], which combines a load cell, accelerometer, and GPS to measure the rowing movement as well as the force exerted by the rower. GPS data obtained from a smartphone are used as the interface and data fusion point. Another example is the system by Prétot et al. [28], which combines two IMUs (accelerometer and gyroscope) with strain gauges. It uses a GoPro for velocity measurement, with strain gauges on the paddle shaft.

Commercially available sensors are still widely used in most studies, and their use is more pronounced in canoeing. One possible explanation is the precision these sensors offer. 

This approach involves synthesizing and integrating existing data points to create new variables, providing a more comprehensive understanding of the data [6]. Researchers employ this method to uncover complex relationships, interactions, or composite measures that enhance the analysis. The derivation of new variables through data synthesis contributes to a deeper and more nuanced analysis of the data presented in the articles [21,26].

### 4.3. Discussion on Data Transmission, Processing and Visualization

The advent of wireless data transmission technologies, such as radio modems, ANT+, and Bluetooth, has significantly enhanced the flexibility and efficiency of data collection in sports performance monitoring systems. This advancement facilitates dynamic and immediate analysis of athlete performance, a critical aspect in the fast-paced environments of canoeing and rowing. However, the range limitations of these technologies pose challenges in open and aquatic settings, where consistent data transmission is crucial for real-time monitoring and feedback.

Only two manuscripts did not report the sampling frequency of the devices used [6,24]. The sampling frequency varied significantly due to the variety of sensors used, but the most common frequency observed was 50 Hz. The choice of frequencies was justified in the manuscripts, explaining that the sensors’ capacities often limited them. Some sensors had low sampling capacities for measuring paddling speed, which necessitated the use of components that could amplify the sampling rate [6,25,26,27,28].

Regarding data storage, some systems recorded the sensor output data on SD cards [28], computers [25,26,27], tablets [26], or mobile devices [6,26]. Other systems utilized microprocessors [24], websites [23] or proprietary software [20,21,22]. One manuscript did not provide information on how the data was stored [19].

In some cases, the data were visualized and analyzed after the tests [22,25,26,27]. However, as shown in Table 4, some studies presented devices allowing for the real-time display and analysis of the data, with the possibility of later downloading additional parameters [19,20,21,23,24]. Two manuscripts did not provide information about data visualization [6,28].

Specifically, Bluetooth is valued for its energy efficiency and reliable point-to-point connections, making it ideal for wearable devices and applications requiring proximity to the athlete. Wi-fi stands out for its ability to support higher bandwidth and range, which is suitable for scenarios that demand the transmission of large volumes of data. The ANT+ protocol is recognized for its effectiveness in creating low-energy sensor networks, which are ideal for complex monitoring systems that require synchronization between multiple devices [32].

Introducing LoRa (Long Range) technology in sports monitoring systems presents a promising avenue to overcome the range limitations of conventional wireless technologies. Its capability for long-range data transmission with minimal power consumption paves the way for more reliable and extensive performance monitoring [33,34].

The dichotomy between devices offering real-time feedback and those necessitating post-analysis reflects divergent approaches to performance analysis. Real-time feedback is invaluable in training scenarios, allowing for immediate adjustments. At the same time, post-analysis provides a platform for a comprehensive evaluation of performance, which is essential for strategic planning and long-term development [35].

Moreover, the intricacies of data storage and variability in sampling frequencies underscore the complex nature of sports performance monitoring. The diversity in data storage solutions reflects the technological evolution and the necessity for adaptable data management strategies that can withstand the environmental challenges inherent in water sports [16].

Integrating emerging technologies like artificial intelligence and the Internet of Things (IoT) promises to transform sports monitoring systems. These technologies can provide deeper, predictive insights into sports performance, moving beyond descriptive analytics to offer prescriptive and personalized athlete feedback [11].

Discussing these elements within the context of sports performance monitoring highlights the current technological capabilities and challenges and sets the stage for future innovations. As the field progresses, developing more sophisticated monitoring systems must consider the balance between technological advancements and the practical needs of athletes, coaches, and researchers, ensuring that these systems are scientifically robust and practically applicable in enhancing athlete performance and training strategies.

The software used for data analysis was Excel [20,22] and MATLAB [23,26,27]. However, other devices already had dedicated software [21]. The reviewed studies designed data analysis processes to extract insights from complex datasets. MATLAB was a mainstay across several studies, facilitating the development of custom routines for data smoothing using low-pass Butterworth filters and identifying specific phases of the athletes’ movements, for example, in the study by Gomes et al. [27] MATLAB was used to automate the detection of the water phase in each stroke, and statistical tests were implemented in SPSS to determine the influence of stroke rates on various performance metrics. As in the study by Bonaiuto et al. [23], MATLAB was used to improve the analysis by automatically detecting strokes and computing essential parameters such as time values and forces.

Furthermore, in the research by Castro et al. [6], a comprehensive approach was taken by combining multiple software tools. Excel was used for data transformation, MATLAB was used to process detailed performance metrics, and R-Studio was used to execute advanced statistical analysis. This integrated use of software platforms enabled a robust and nuanced interpretation of the data, ensuring the reliability of the findings. Holt et al. [22] demonstrated the effective use of the Statistical Analysis System (SAS) for advanced statistical modeling in sports performance research, alongside Excel’s utility in data alignment and preprocessing.

It must be noted that some of the devices used are commercialized and that the information offered may be limited.

### 4.4. Limitations on Review

When interpreting the manuscripts in this review, certain limitations should be considered. The exclusion of gray literature may have led to the omission of studies that had not undergone formal publication. Furthermore, only manuscripts written in English were included, potentially excluding relevant research published in other languages.

## 5. Conclusions

The primary contribution of this systematic review is the comprehensive evaluation of sensor technologies used in water sports, particularly rowing and canoeing. By synthesizing data from various studies, this review highlights the significant advancements in applying inertial, force, and position sensors for measuring key performance parameters. These technologies may provide valuable data that enhance training strategies, monitor athlete performance, and aid in injury prevention.

Moreover, this review identifies critical challenges, such as variability in sampling frequencies and data storage solutions, emphasizing the need for standardized protocols. It also underscores the potential of emerging technologies like artificial intelligence and the Internet of Things to transform sports performance monitoring by offering deeper predictive insights and more personalized feedback.

The variability in sensor technology and methodologies across the included studies underscores the need for standardized protocols to enhance the comparability of future research. Future research efforts should prioritize the development of standardized sensor protocols and data collection methods. Such efforts will improve the comparability of findings across studies and support the establishment of best practices in water sports biomechanical monitoring.

This systematic review offers a detailed overview of current sensor technologies, their applications, and future directions. It provides a foundation for further research and development to optimize athlete performance and safety in water sports.

## Figures and Tables

**Table 1:** PECO.

P	Athletes Who Practice Rowing or Canoeing
**E**	Documents describing sensors to monitor or analyze performance in rowing and canoeing
**C**	Do not apply
**O**	Multiple sensors and variables for analysis of the performance in rowing and canoeing

**Table 2:** Assessment of NHLBI.

Author	1	2	3	4	5	6	7	8	9	10	11	12	13	14	Quality Rating
[19]	Y	N	Y	Y	Y	NA	NA	NA	Y	Y	Y	NA	NA	NR	GOOD
[20]	Y	Y	Y	Y	N	NA	NA	NA	Y	Y	Y	NA	NA	NR	GOOD
[21]	Y	Y	Y	Y	N	NA	NA	NA	Y	N	Y	NA	NA	NR	GOOD
[22]	Y	Y	Y	Y	N	NA	NA	NA	Y	N	Y	NA	NA	NR	GOOD
[23]	Y	Y	NA	NR	N	NA	NA	NA	Y	N	Y	NA	NA	NR	GOOD
[24]	Y	NA	NA	NA	NA	NA	NA	NA	Y	NA	Y	NA	NA	NA	FAIR
[25]	Y	Y	Y	Y	N	NA	NA	NA	Y	Y	Y	NA	NA	NR	GOOD
[26]	Y	NA	NA	NA	NA	NA	NA	NA	Y	NA	Y	NA	NA	NA	FAIR
[27]	Y	Y	Y	Y	N	NA	NA	NA	Y	N	Y	NA	NA	NR	GOOD
[6]	Y	NA	NA	NA	NA	NA	NA	NA	Y	NA	Y	NA	NA	NR	FAIR
[28]	Y	Y	Y	Y	N	NA	NA	NA	Y	NR	Y	NA	NA	NR	GOOD

Legend: Y = Yes, N = No, NA = Not Applicable, NR = Not Reported.

**Table 3:** Summary table of variables collected, systems, and sensors used.

Reference	Sport	Parameters	Sensors
[19]	C	Stroke length; Impulse; Peak force; Time to peak force	Kayak Power Meter
[20]	C	Stroke rate; Hand force output; Acceleration; Rotational velocity; Heart rate	Kayak Power Meter
[21]	R	Boat velocity; Stroke rate; Time from catch to minimum velocity; Distance per stroke; Mean force; Power output; Peak force; Time to peak force from the catch; Peak force angle; Gate angle	PeachPowerLine; GPS
[22]	R	Oar angle and force; Boat velocity per stroke	Peach PowerLine GPS
[23]	C	Instantaneous stroke rate; Boat speed; Travelled distance; Angular acceleration; Forces on paddle and footrest; Force impulse; Time to peak; Stroke time; Cycle time	eKayak
[24]	C	Cadence	Vaaka
[25]	R	Boat velocity and acceleration; Horizontal/vertical oar angle; Normal component of the force; Seat position	Electromagnetic impeller; Accelerometer; Potentiometers; Strain gauge
[26]	C	Force in the paddle shaft	Strain gauges; Accelerometer; Gyroscope
[27]	C	Force in the paddle; Acceleration	FPaddle
[6]	R	Force; Acceleration; Speed; Power of stroke	Load cell; Accelerometer; GPS
[28]	C	Force; Velocity	Strain gauges; Accelerometer; Gyrometer; Go pro

Legend: R—Rowing and C—Canoeing.

**Table 4:** Summary table of storage and transmission system, data visualization, and analysis software.

Reference	Sampling Frequency	Storage Systems	Visualization of Data	System of Data Transfer	Software for Data Analysis
[19]	50 Hz	-	Real-time	ANT+ protocol or Bluetooth	-
[20]	1 Hz	Proprietary Software	Real-time	-	Excel (version 16.40) R-Studio (v4.0.2, Boston, FL, USA)
[21]	50 Hz	Proprietary Software	Real-time	-	Proprietary Software
[22]	50 Hz	Proprietary Software	Not real time	-	Excel SAS (version 9.4, SAS Institute, Cary, NC, USA)
[23]	50 Hz	Website	Real-time	Wi-fi	MATLAB (version R2018b by The MathWorks Inc., Natick, MA, USA)
[24]	-	Microprocessor	Real-time	ANT+ protocol	Proprietary Software
[25]	25 Hz	PC	Not real time	Radio Modems	-
[26]	100 Hz	Mobile phone, tablet, and PC	Not real time	Radio Modems	MATLAB (version R2011a) (The MathWorks Inc., Natick, MA, USA)
[27]	256 Hz	PC	Not real time	Radio Modems	MATLAB (version R2010a by The MathWorks Inc., Natick, MA, USA) IBM SPSS (version 12.0, IBM Inc., Chicago, IL, USA)
[6]	-	Mobile device	-	Bluetooth	-
[28]	100 Hz	SD Card	-	Bluetooth	-

Legend: ANT—Advanced and Adaptive Network Technology, PC—Personal Computer, and SD—Secure Digital.

**Table 5:** Summary table of critical sensors: measurable variables and applications in canoeing and rowing.

Sensor Type	Measurable Variables	Applications in Canoeing and Rowing
Accelerometer	Angular position	Monitoring stroke acceleration, boat movement patterns, and paddle dynamics
Electromagnetic Impeller	Boat Velocity and acceleration	Measuring boat dynamics
Global Position System	Position, Speed, and Distance	Tracking boat velocity, measuring distance covered, race pacing strategies
Gyroscope	Angular velocity and orientation	Analyzing rotational movement and stability during paddling
Load Cell	Force and Weight	Measuring force exerted on the paddle
Potentiometer	Angular Position	Measuring our angles during strokes
Strain Gauges	Force and Pressure	Analyzing force distribution on paddles

## Appendix A

**Table A1:** Searched databases and associated search terms used.

Database	Search Terms
Web of Science (Core collection)	“athletes rowing” OR “athletes canoeing” OR canoeing OR rowing OR kayak (Title) and “inertial sensor” OR sensor OR gyroscope OR accelerometer OR “strain gauge” OR GPS OR “Global Position System” OR GNSS OR “Global Navigation Satellite System” (Abstract) and kinetic OR kinematics OR velocity OR power OR time OR force OR angle OR biofeedback (Topic) not ergometer (Topic)
Scopus	(TITLE ((“athlete$ row*” OR “athletes canoe*” OR canoeing OR rowing OR kayak)) AND ABS ((“inertial sensor$” OR sensor$ OR gyroscope OR *meter OR “strain gauge$” OR GPS OR “Global Position System” OR GNSS OR “Global Navigation Satellite System”)) AND TITLE-ABS-KEY ((kine* OR velocity OR power O tim* OR force$ OR angle$ OR *feedback)) AND NOT TITLE-ABS-KEY (ergometer))
Science Direct	(TITLE ((“athlete rowing” OR “athletes canoeing” OR canoeing OR rowing OR kayak)) AND TITLE-ABS-KEY ((“inertial sensor” OR sensor OR gyroscope OR accelerometer OR “strain gauge” OR GPS OR “Global Position System” OR GNSS OR “Global Navigation Satellite System” OR kinetic OR kinetics OR velocity OR power OR time OR force OR angle OR biofeedback)) AND NOT TITLE-ABS-KEY (ergometer))
Sage Journals	Anywhere (kine* OR velocity OR power OR tim* OR force$ OR angle$ OR *feedback) AND NOT Anywhere (ergometer) AND Abstract (“inertial sensor$” OR sensor$ OR gyroscope OR *meter OR “strain gauge$” OR GPS OR “Global Position System” OR GNSS OR “Global Navigation Satellite System”) AND Title (“athlete$ row*” OR “athletes canoe*” OR canoeing OR rowing OR kayak)

## References

[1] Buckeridge E.M., Bull A.M.J., McGregor A.H. (2015). Biomechanical Determinants of Elite Rowing Technique and Performance. Scand. J. Med. Sci. Sport..

[2] Xiao G., Zheng W.T. (2013). Development of Canoe Equipments for Strength Training in Rowing Tank. Adv. Mater. Res..

[3] Wu Y., Xu G. (2019). Monitoring the Physiological and Biochemical Indicators of Teenage Male Rowers during Winter Training. IOP Conf. Ser. Earth Environ. Sci..

[4] Hendelman D.L., Whittlesey S., Caldwell G.E., Freedson P.S. (1998). Biomechanical and Physiological Determinants of Rowing Performance. Med. Sci. Sport. Exerc..

[5] Nurkholis, Mintarto E., Hariyanto A. (2019). Physical and Psychological Indicators for Canoeing Athlete Selection. J. Adv. Soc. Sci. Humanit..

[6] Castro R., Mujica G., Portilla J. (2022). Internet of Things in Sport Training: Application of a Rowing Propulsion Monitoring System. IEEE Internet Things J..

[7] Baca A., Kornfeind P., Heller M., Moritz E.F., Haake S. (2006). Feedback Systems in Rowing. Proceedings of the Engineering of Sport 6.

[8] Mäestu J., Jürimäe J., Jürimäe T. (2005). Monitoring of Performance and Training in Rowing. Sport. Med..

[9] Klitgaard K.K., Hauge C., Oliveira A.S., Heinen F. (2021). A Kinematic Comparison of On-Ergometer and on-Water Kayaking. Eur. J. Sport. Sci..

[10] Mendes J.J.A., Vieira M.E.M., Pires M.B., Stevan S.L. (2016). Sensor Fusion and Smart Sensor in Sports and Biomedical Applications. Sensors.

[11] Camomilla V., Bergamini E., Fantozzi S., Vannozzi G. (2018). Trends Supporting the In-Field Use of Wearable Inertial Sensors for Sport Performance Evaluation: A Systematic Review. Sensors.

[12] Worsey M.T.O., Espinosa H.G., Shepherd J.B., Thiel D.V. (2019). Inertial Sensors for Performance Analysis in Combat Sports: A Systematic Review. Sports.

[13] Espinosa H.G., Shepherd J.B., Thiel D.V., Worsey M.T.O. (2019). Anytime, Anywhere! Inertial Sensors Monitor Sports Performance. IEEE Potentials.

[14] McNab T., James D.A., Rowlands D. (2011). IPhone Sensor Platforms: Applications to Sports Monitoring. Proceedings of the Procedia Engineering.

[15] Worsey M.T.O., Espinosa H.G., Shepherd J.B., Thiel D.V. (2019). A Systematic Review of Performance Analysis in Rowing Using Inertial Sensors. Electronics.

[16] Liu L., Qiu S., Wang Z.L., Li J., Wang J.X. (2020). Canoeing Motion Tracking and Analysis via Multi-Sensors Fusion. Sensors.

[17] Peltonen J., Rusko H. (1993). Interrelations between Power, Force Production and Energy Metabolism in Maximal Leg Work Using a Modified Rowing Ergometer. J. Sport. Sci..

[18] Rethlefsen M.L., Kirtley S., Waffenschmidt S., Ayala A.P., Moher D., Page M.J., Koffel J.B., Blunt H., Brigham T., Chang S. (2021). PRISMA-S: An Extension to the PRISMA Statement for Reporting Literature Searches in Systematic Reviews. Syst. Rev..

[19] Macdermid P.W., Gilbert C., Jayes J. (2020). Using a Kayak Paddle Power-Meter in the Sport of Whitewater Slalom. J. Hum. Sport. Exerc..

[20] Hogan C., Binnie M.J., Doyle M., Peeling P. (2022). Quantifying Sprint Kayak Training on a Flowing River: Exploring the Utility of Novel Power Measures and Its Relationship to Measures of Relative Boat Speed. Eur. J. Sport. Sci..

[21] Holt A.C., Aughey R.J., Ball K., Hopkins W.G., Siegel R. (2020). Technical Determinants of On-Water Rowing Performance. Front. Sport. Act. Living.

[22] Holt A.C., Siegel R., Ball K., Hopkins W.G., Aughey R.J. (2022). Prediction of 2000-m on-Water Rowing Performance with Measures Derived from Instrumented Boats. Scand. J. Med. Sci. Sport..

[23] Bonaiuto V., Gatta G., Romagnoli C., Boatto P., Lanotte N., Annino G. (2020). A Pilot Study on the E-Kayak System: A Wireless DAQ Suited for Performance Analysis in Flatwater Sprint Kayaks. Sensors.

[24] Croft H., Ribeiro D.C. (2013). Developing and Applying a Tri-Axial Accelerometer Sensor for Measuring Real Time Kayak Cadence. Proceedings of the Procedia Engineering.

[25] Kleshnev V. (2010). Boat Acceleration, Temporal Structure of the Stroke Cycle, and Effectiveness in Rowing. Proc. Inst. Mech. Eng. P J. Sport. Eng. Technol..

[26] Sturm D., Yousaf K., Brodin L.Å., Halvorsen K. (2013). Wireless Kayak On-Water Ergometry—Part 1: Paddle Blade Force. Sport. Technol..

[27] Gomes B.B., Ramos N.V., Conceição F.A.V., Sanders R.H., Vaz M.A.P., Vilas-Boas J.P. (2015). Paddling Force Profiles at Different Stroke Rates in Elite Sprint Kayaking. J. Appl. Biomech..

[28] Prétot C., Carmigniani R., Hasbroucq L., Labbé R., Boucher J.P., Clanet C. (2022). On the Physics of Kayaking. Appl. Sci..

[29] Checking out the Kayak Power Meter System|DC Rainmaker. https://www.dcrainmaker.com/2013/06/checking-power-system.html.

[30] Peach Innovations—Rowing Telemetry and Instrumentation. http://www.peachinnovations.com/.

[31] Vaaka Paddle Cadence Sensor—Vaaka Cadence. https://www.vaakacadence.com/shop/vaaka-paddle-cadence-sensor/.

[32] Tosi J., Taffoni F., Santacatterina M., Sannino R., Formica D. (2017). Performance Evaluation of Bluetooth Low Energy: A Systematic Review. Sensors.

[33] Augustin A., Yi J., Clausen T., Townsley W. (2016). A Study of LoRa: Long Range & Low Power Networks for the Internet of Things. Sensors.

[34] Cattani M., Boano C., Römer K. (2017). An Experimental Evaluation of the Reliability of LoRa Long-Range Low-Power Wireless Communication. J. Sens. Actuator Netw..

[35] Rajšp A., Fister I. (2020). A Systematic Literature Review of Intelligent Data Analysis Methods for Smart Sport Training. Appl. Sci..

[36] Page M.J., McKenzie J.E., Bossuyt P.M., Boutron I., Hoffmann T.C., Mulrow C.D., Shamseer L., Tetzlaff J.M., Akl E.A., Brennan S.E. (2021). The PRISMA 2020 statement: An Updated Guideline for Reporting Systematic Reviews. Br. Med. J..

